# Virtual Reality as a Therapeutic Tool in Spinal Cord Injury Rehabilitation: A Comprehensive Evaluation and Systematic Review

**DOI:** 10.3390/jcm13185429

**Published:** 2024-09-13

**Authors:** Matteo Scalise, Tevfik Serhan Bora, Chiara Zancanella, Adrian Safa, Roberto Stefini, Delia Cannizzaro

**Affiliations:** 1 Faculty of Medicine and Surgery, Vita-Salute San Raffaele University, Via Olgettina 58, 20132 Milan, Italy; 2 Department of Molecular Medicine, University of Pavia, Via Forlanini 14, 27100 Pavia, Italy; 3 Department of Neurosurgery, Mayo Clinic Florida, Scottsdale, AZ 85259, USA; 4 Department of Neurosurgery, ASST Ovest Milano Legnano Hospital, Via Papa Giovanni Paolo II, 20025 Legnano, Italy

**Keywords:** physical therapy, rehabilitation, spinal cord injury, spine, virtual reality

## Abstract

**Introduction**: The spinal rehabilitation process plays a crucial role in SCI patients’ lives, and recent developments in VR have the potential to efficiently engage SCI patients in therapeutic activities and promote neuroplasticity. **Objective**: The primary objective of this study is to assess a complete review of the extended impacts of VR-assisted training on spine rehabilitation in SCI patients. **Methods**: This systematic review was conducted according to Preferred Reporting Items for Systematic Reviews and Meta-Analyses (PRISMA) through a single database search in PubMed/Medline between the dates 1 January 2010 and 1 February 2024. MESH terms and keywords were combined in the following search strategy: (Augmented Reality OR VR OR Virtual Reality) AND (Spine OR Spinal) AND Rehabilitation. Included articles were written in English, involved adults with SCI, included an intervention with VR, AR, or any mixed reality system, and assessed changes in outcomes after the intervention. **Results**: The search produced 257 articles, and 46 of them were allocated for data extraction to evaluate 652 patients. Both when VR training was analyzed and reviewed separately, and when compared to traditional training, the findings exhibited predominantly promising outcomes, reflecting a favorable trend in the study. VR technologies were used in different settings and customizations, and the medium total time of VR training among the studies was 60.46 h per patient. **Conclusions**: This auspicious outcome of the study further motivates the intervention of VR and AR in the rehabilitation of SCI patients along with ameliorating their overall holistic well-being.

## 1. Introduction

The trajectory of virtual reality (VR) technology, from its inception as a concept to its present-day implementation across various sectors such as healthcare and rehabilitation, has been nothing short of impressive [1]. Though initially created for entertainment purposes like gaming, VR has transformed into an influential therapeutic tool currently used in the medical field, with the potential to bring about life-altering changes for selected patients, especially in the field of spinal cord injury (SCI) rehabilitation [2]. Virtual reality (VR) is still in the early stages of integration into clinical practice, with several barriers slowing its adoption. Key challenges include a lack of time and expertise on how to use VR in treatment, a lack of personalization of some VR applications to patient needs and treatment goals, or a gap in knowledge on the added value of VR in a specific setting. For these technologies to achieve their intended impact, they must be seamlessly integrated into existing healthcare practices and aligned with the needs of patients and healthcare providers [1]. Throughout history, spinal cord injuries have presented considerable hurdles for both patients and healthcare providers, which have frequently led to persistent disability and decreased quality of life [3,4]. Conventional methods used in rehabilitation efforts were moderately successful but failed to address the intricate physical and cognitive challenges that accompany SCI completely [5]. Nevertheless, with the emergence of VR technology, new opportunities exist for improving rehabilitative outcomes while helping those affected by SCI attain greater functional autonomy that enhances their overall health and wellness. VR-based interventions have become increasingly popular in recent years as a means of enhancing traditional rehabilitation techniques for patients with SCI. These cutting-edge approaches utilize the interactive qualities of VR environments to engage patients in personalized exercises, activities, and simulations that specifically target motor skills, sensory abilities, and cognitive functions [6]. By creating realistic situations and delivering immediate feedback, this technology enables individuals dealing with an SCI to safely hone their movements while improving muscle strength and coordination as well as overall sensorimotor integration capabilities [7].

The utilization of virtual reality technology in spinal cord injury rehabilitation is diverse and includes a range of interventions, with assessed benefits that range from motor learning and gait balance to pain management and psychological well-being [8,9]. Patients are immersed in simulated virtual environments through head-mounted displays with motion-tracking sensors, which replicate everyday tasks like walking, reaching, or navigating obstacles [10]. For patients requiring flexibility towards their levels of sensory input or interaction, fully immersive, semi-immersive as well as non-immersive VR setups utilizing screens or projections can be useful alternative approaches. Incorporating VR technology with advanced innovations such as body–machine interface (BMI) and brain–computer interface (BCI) systems not only broadens the scope of customized rehabilitation interventions for SCI patients but also presents new avenues to enhance their recovery [11]. The ability to harness neural signals or brain activity towards controlling virtual avatars or prosthetic devices holds significant potential in improving motor functionality, neuroplasticity, and functional autonomy among individuals suffering from severe spinal cord injuries [12]. Although VR’s potential in SCI rehabilitation is increasingly recognized, more systematic evaluation and synthesis of existing evidence are necessary to determine its efficacy, feasibility, and clinical relevance.

The objective of this systematic review is to conduct a thorough examination and evaluation of the available literature to extensively investigate the effects of virtual reality as a therapeutic tool in spinal cord injury rehabilitation.

## 2. Methods

This systematic review was conducted according to Preferred Reporting Items for Systematic Reviews and Meta-Analyses (PRISMA) [13]. The review was not registered.

### 2.1. Search Strategy

A systematic search was performed in a single database (PubMed/Medline) up to the 1st of March 2024. Articles published from 1 January 2010 to 1 February 2024 were included in the search. MESH terms and keywords were combined in the following search strategy: (Augmented Reality OR VR OR Virtual Reality) AND (Spine OR Spinal) AND Rehabilitation. A language filter was applied to include only articles written in English.

### 2.2. Eligibility Criteria

The inclusion criteria of the selected studies were as follows: (1) written in English, (2) comprised adults (>18 years old) with SCI, (3) included an intervention with VR, augmented reality (AR), or any mixed reality system, and (4) assessed changes in outcomes after the intervention.

All study designs were deemed eligible for inclusion, except for reviews or meta-analyses.

Three reviewers (M.S., T.S.B., and C.Z.) independently screened articles for inclusion, extracted data, and evaluated the methodological quality of the trials. Conflicts were resolved by a senior author.

### 2.3. Data Extraction

The data were entered in a customized Excel^®^ (Version 16.89) spreadsheet. Data included studies’ characteristics (authors, time of publication, continent/country, and journal of publication); methodological details (study design, type of VR intervention, and duration of VR training as hours/week, hours/month, and total hours); and information on participants characteristics, VR effects, and outcome measurements. 

Overall, this review comprises studies in which VR system training was compared to traditional training, as well as studies in which outcomes were assessed after patients underwent “VR training” or “VR + BMI/BCI” or “VR + conventional therapy” (with no direct comparison to traditional training).

### 2.4. Risk of Bias Assessment

This study was thoroughly assessed for potential sources of bias that could impact the validity of its findings. Three independent reviewers (M.S., T.S.B., and C.Z.) examined the articles, with each article being reviewed by a different individual. Any discrepancies were resolved by senior reviewers (A.S. and D.C.). 

No specific tools were used to assess the methodological quality of the studies. 

Although each analyzed study had errors, they could not be addressed or corrected. The cumulative risks associated with individual extractions could not be resolved.

## 3. Results

### 3.1. Selection of Studies

The search produced 257 articles. In total, 205 were excluded due to unmatching titles or abstracts. A total of 52 underwent full-text review, and 6 articles were excluded for either wrong intervention (n = 2), wrong settings (n = 3), or wrong population (n = 1). In conclusion, 46 articles fulfilled the inclusion criteria and were selected for the systematic review.

**Figure 1:**
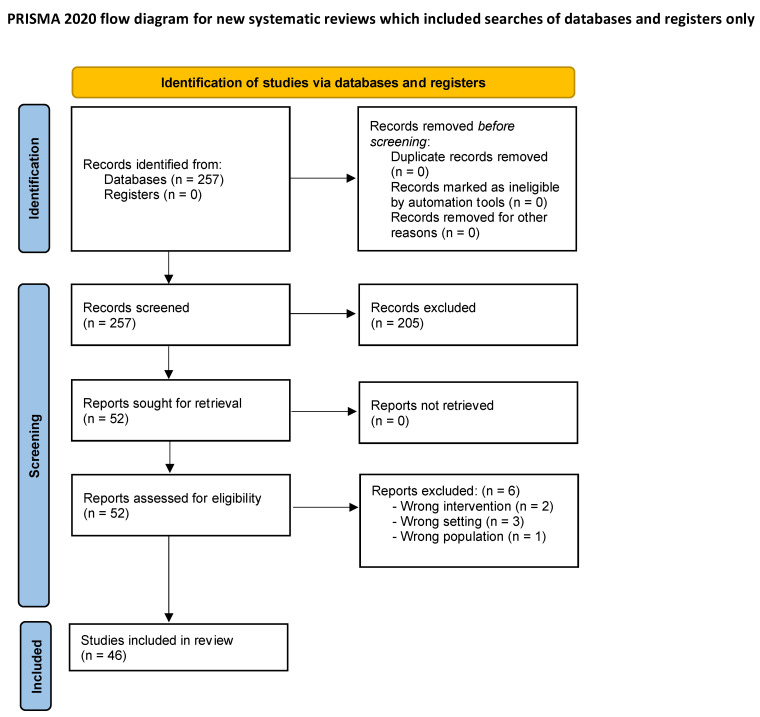
PRISMA flow diagram for systematic reviews.

Figure 1 shows the PRISMA 2020 flow diagram for systematic reviews with the selection process of studies identified and included.

### 3.2. Participant Characteristics

Data from the 46 articles were extracted, and, considering only patients with evaluation of outcome data, the sample included 652 patients.

A majority of 478 were men, 162 were women, and the sex of about 12 patients was not specified. (478 M; 162 F; 12 not specified (n.s.)) 

The mean age of patients who underwent intervention was 44.47 and ranged greatly between different studies spanning from 23 to 60 years old (41.5 ± 18.5).

The American Spinal Injury Association (ASIA) impairment scale or AIS [14] was used to categorize patients based on their SCI type. 

Table 1 contains all the information on the characteristics of the studies, including patients’ characteristics.

### 3.3. Design of the Studies

Among the 46 studies analyzed, 23 are clinical trials, 6 are case reports, 8 have a pretest–posttest design, 1 has an interrupted time series design, 1 has a concurrent nested design, 1 is a longitudinal pilot study, 1 is a correlational study, 1 is a case–control study, 1 is a single-arm study of neuroimaging data, 1 is a preliminary study, and 2 have experimental designs that were not specified.

Another aspect that deserves attention is the sample size, which ranged from 1 to 59 participants. 

### 3.4. VR Characteristics

VR technologies used in different settings and studies can be categorized into immersive VR (18 articles), semi-immersive VR (10 articles), non-immersive VR (13 articles), and a combination of VR and BMI/BCI systems (5 articles).

### 3.5. Time of Training

Duration of the training was considered as hours/week, hours/month, and total hours per patient. The medium total time of VR training among the studies was 60.44 h per patient. This average is calculated among 40 articles of the 46 included: 6 studies did not specify the total hours of training for patients (the names being Al Nattah et al., 2024 [16], Casadio et al., 2011 [20], Putrino et al., 2021 [45], Jordan et al., 2016 [55], Trincado-Alonso et al., 2014 [59], and Tamplin et al., 2020 [60]). Among the considered studies, two present with notable outliers compared to the others: Donati et al., 2016 [54] had a total training time of 1958 h while Miguel A.L. Nicolelis et al., 2022 [36] reported a total training time of 118 h, where the median value is 12 h of training. 

The intervals between VR-based interventions varied between studies, with a frequency ranging from two to five times a week, with sessions lasting from 20 to 150 min. However, some studies did not clearly report the number of sessions.

### 3.6. Outcome Measures

In the selected studies, a wide variety of outcome measures were assessed. To analyze the results, they have been categorized into five different subgroups: (1) upper limb mobility/strength; (2) lower limb mobility, postural stability, gait and walking, and sitting balance; (3) quality of life, daily life independence, functional/cognitive ability, and psychological outcomes; (4) pain; and (5) other outcome measures and feasibility of the VR system.

Further details about the scales used to assess VR use’s outcomes can be found in the respective tables.

Considering the whole five subgroups of the outcome measures, the analysis/review demonstrated overall positive results of VR training both compared to traditional training and considered independently. 

Few of the studies found no statistically significant difference in outcomes between VR and traditional systems, but no article assessed inferior results for VR compared to traditional training. Even when there was no statistically significant difference in outcomes between VR and conventional therapy, there were still better results in the experimental group (EG) compared to the control group (CG), only not enough to be considered statistically significant, which could be related to the population of patients selected by the studies. 

To ensure a clearer understanding of these results, studies comparing VR training with traditional therapy will be labeled as “comparative studies”, in both the written content and tables.

**Upper limb mobility and strength** were evaluated in seven studies through 12 different outcome scales, with overall positive results of VR training both compared to traditional training and considered individually. Few of the studies found no significant difference in upper limb (UL) outcomes between VR and traditional systems, but no article assessed inferior results for VR compared to traditional training. Taking comparative studies into consideration, 27% of the tests defined a better outcome compared to traditional training, especially when “muscle strength” was tested.

In studies without a comparison group, 100% of tests saw a significant improvement related to VR training when compared to baseline.

All specific data are outlined in Table 2.

**Lower limb mobility, gait and walking, postural stability, and balance** were evaluated in 18 studies through 20 different outcomes. Even for lower limbs (LLs), the overall results were positive, both comparing VR to traditional training and considering VR individually. None of the studies, however, demonstrated an inferior performance of VR systems for the explored purposes. 

Focusing on comparative studies, 71% of the tests defined a better outcome compared to traditional training. 

In the other studies, 80% of scales saw a significant improvement with VR training when compared to baseline.

All specific data are outlined in Table 3.

**Quality of life, daily life independence, functional/cognitive ability, and psychological outcomes** were evaluated in 17 studies through 21 different outcome scales. In total, 10 studies were comparative ones, and 56% of the tests analyzed found a statistically significant improvement in the assessed measures compared to the control group. The remaining 7 articles reported outstanding results, with 100% of the tests improving after VR training compared to baseline. 

All specific data are outlined in Table 4.

**Pain** was evaluated in 12 studies through seven different outcome scales. Among these studies, 3 articles compared the efficacy of VR systems in pain management with control groups and found a significant improvement in 100% of tests. The remaining 9 articles with no control groups demonstrated positive results in pain control after the use of VR in their experimental group across 66.6% of tests. 

All specific data are outlined in Table 5.

**Other outcome measures and feasibility of the VR system:** The last subgroup comprises scales assessing various outcomes such as the feasibility of the VR system, changes in body ownership, perceived exertion using the system, changes in sensory parameters, absence of side effects, improvement of driving abilities, improved effectiveness of BCI, use of VR in diagnostics and assessment, perceived interaction and immersion, and motor imagery. These have been grouped into 14 different groups of parameters, ranging from the feasibility of the VR system to side effects of VR use and quality of the perceived VR environment, changes in body ownership and changes in sensory parameters, brain/brainstem plasticity, and improvement in driving abilities after using a dedicated simulator. 

Two of these studies assessed the respective outcomes with the comparison with a control group, and better results in VR-assisted training were found across 100% of criteria, encouraging the use of VR systems. Twelve studies assessed the experimental group, without a control group, across various parameters: in 93.3% of cases, the use of VR systems had a positive impact on the outcome measured. Notably, the only negative result, reported by Pozeg et al., 2017 [50], is merely an absence of significant improvement in one of the outcomes measured (full body illusion) after the intervention.

All specific data are outlined in Table 6.

## 4. Discussion

The current literature comprises different reviews on the examined topic; however, this is the first of its kind to refine a holistic analysis comprising all outcomes associated with VR rehabilitation. We indeed take into consideration a whole range of different benefits and changes related to the rehabilitation: upper limb mobility/strength; lower limb mobility, postural stability, gait and walking, and sitting balance; quality of life, daily life independence, functional/cognitive ability, and psychological outcome; pain; and other outcome measures and feasibility of the VR system. Upper and lower limb mobility and strength are surely the most analyzed outcomes in previous reviews [7,34,61,62,63,64], together with pain changes [41,65]. In this review we try to implement different and new points of view, analyzing outcomes in quality of life, psychological well-being, and variables that measure the feasibility of different VR systems.

The introduction of VR technology in spinal rehabilitation is a big step from traditional methods. Conventional treatments are moderately successful but do not adequately cater to the complex cognitive and physical challenges associated with SCI like VR-based interventions do. Unlike them, these types of therapy are more holistic and interesting, thus improving the outcomes of rehabilitation as well as functional independence among patients. Each of the outcomes was categorized into one of the five different subgroups and fully analyzed.

### 4.1. Upper Limb Mobility and Strength

For upper limb rehabilitation, setting VR and traditional training side by side, this review shows confident results mostly when “Muscle Strength” was studied, with 100% of the related scales showing better outcomes for VR. On the contrary, “Hand dexterity” measures showed no valuable difference between the two training types.

When taking the duration of training into account, we noticed that patients with a higher total number of training hours (especially when higher than 15 h) underwent better outcomes overall compared to patients with shorter periods of rehabilitation. This emphasizes the need, particularly for mobility and strength effects, to set a higher baseline as standard hours of VR training.

### 4.2. Lower Limb Mobility, Postural Stability, Gait and Walking, and Sitting Balance

The second group was the most represented one of all, with 18 studies evaluating lower limbs’ strength together with physical mobility and balance. A highly positive trend was observed when analyzing data from these studies, and this probably depicts one of the best results shown, especially considering that body mobility and balance are among the outcomes patients prioritize the most.

Among the comparative studies, only one (Sengupta et al., 2020 [25]) assessed no significant difference in lower limb outcomes between VR and conventional therapy; however, the *p* value for this assessment being *p* = 0.001 is surely a limitation to consider.

### 4.3. Quality of Life, Daily Life Independence, Functional/Cognitive Ability, and Psychological Outcomes

The general quality of life improvements constitute the starting point when assessing a rehabilitation program; in this review, we tried to implement and consider all the different aspects that influence a person’s well-being other than mobility and strength. “Daily life Independence” is surely one of the main factors to consider; on this topic, our evaluation displays quite optimistic results, enhancing the potential role of VR training in restoring dignity and liberty for patients with SCI.

A quite new point of view analyzed here is represented by the “Psychological changes and outcomes” related to rehabilitation. Patients with SCI often suffer from mild to severe types of mood disorders, with depression or depressive-like behaviors being the most common; it becomes fundamental at this point to try and observe the differences between disparate kinds of training in influencing patients’ mood. The potential benefits of VR training in enhancing the psychological well-being of SCI patients are mainly related to the higher level of enjoyment shown when compared to traditional training, especially when immersive or semi-immersive settings are used. On top of that, game-like systems are frequently incorporated in VR exercises, and this firmly contributes to the positive impact of training on patients’ spirits.

### 4.4. Pain

Taking comparative studies into consideration, the “Pain” measure had the most astonishing results, with 100% of scales assessing a better outcome for VR settings compared to traditional ones. The three most used scales to assess neuropathic pain were the Neuropathic pain intensity Numeric Rating Scale (NRS), the Neuropathic Pain Scale (NPS), and the Visual Analog Scale (VAS), and all three of them revealed significant improvements in most of the studies, and also when comparing VR training alone to the baseline. When looking at the articles that considered pain variables as outcomes, the duration of training varies significantly, from 0.05 to 13.5 total hours, though it does not result in a consistent related difference in results. Pain reduction remains another cardinal goal of rehabilitation, with these results thus strengthening the position of VR settings in current recovery programs.

### 4.5. Other Outcome Measures and Feasibility of the VR System

One more novelty of this review stands in reassessing a whole variety of different outcomes, seemingly unrelated, but quite important when assessing the feasibility of the usage of VR systems in rehabilitation programs. Through gathering these various scales, the main purpose was to report the level of practicality and viability of these technologies, with current results that show an overwhelmingly positive trend.

### 4.6. Limitations of the Study

The application of VR in SCI rehabilitation still has some drawbacks despite its positive results. One major point is that different studies used various VR systems and rehabilitation protocols producing issues with treatment standardization and outcome comparison. Another limitation lies in the time taken for each session, which ranges from 20 to 150 min, and training frequency, fluctuating from 2 to 5 times/week. This makes it challenging to define what is the best amount of VR therapy that one needs to achieve maximum benefits. Besides this, cost implications together with the need for specialized training among healthcare providers act as barriers to achieving widespread implementation.

VR-based rehabilitation is estimated to be more effective and accessible in future developments. Artificial intelligence (AI) systems with machine learning (ML) algorithms can provide custom adaptive programs working together with virtual reality. Real-time patient performance tracking abilities of this technology allow for adjusting task complexity levels to always keep the patients at the highest possible challenge point to maximize therapeutic gains. This can be analogized with neuromonitoring that has been commonly used during neurosurgery, and these advanced VR methodologies in question also have the potential to benefit patients who had tumor-resection surgeries and need rehabilitation post-operatively [66]. Portable wearable devices combined with wireless sensors might also enhance convenience when using VR systems for home-based recovery purposes. This transition would be very beneficial, especially for those individuals who cannot move around freely due to limited mobility or living far away from hospitals. Also, it could be even more effective if augmented reality (AR) was used alongside virtual reality in creating mixed-reality environments, where patients can seamlessly shift between tasks completed within these two worlds. If such integration happened, then skills acquired through simulated environments would easily transfer into real-life situations thereby further improving functional outcomes.

## 5. Conclusions

The use of VR has the potential to be a game-changer for SCI rehabilitation. It increases motor and cognitive functions better than traditional methods, particularly when used in conjunction with other non-invasive treatments. In saying that, there are still some problems that need to be solved before it can be widely adopted such as the inconsistency between VR systems and protocols, the duration of sessions, the frequency of sessions, and high costs that make standardization difficult. In the future, we may see more personalized adaptive programs that integrate AI, ML, and wearable technology thereby enhancing therapy outcomes through real-time adjustment of task difficulty based on patient performance.

## Figures and Tables

**Table 1:** Characteristics of the studies, including patients’ characteristics (age, gender, and level of injury).

Studies	Study Design	Sample (n)	Mean Age (Years)	M	F	AIS Grade [14]
Lim et al., 2020 [15]	clinical trial	20	59.0	14	6	C-D
Al Nattah et al., 2024 [16]	case report	1	23.0	0	1	D
Prasad et al., 2018 [17]	clinical trial	22	23.7	21	1	A-B-C-D
Dimbwadyo-Terrer et al., 2016 [18]	clinical trial	31	34.5	22	9	A-B
Dimbwadyo-Terrer et al., 2016 [19]	clinical trial	9	54.3	7	2	A-D
Casadio et al., 2011 [20]	clinical trial	6	40.1	6	0	A-C
Bressi et al., 2023 [21]	case report	1	23.0	1	0	B
Nair et al., 2022 [22]	clinical trial	21	32.45	13	8	A-B
Goel et al., 2021 [23]	clinical trial	18	39.1	15	3	B-C-D
Manzanares et al., 2021 [24]	pretest–posttest design	11	42.4	7	4	A-C-D
Sengupta et al., 2020 [25]	pretest–posttest design	33	28.0	27	6	A-B-C-D
Khurana et al., 2017 [26]	pretest–posttest design	30	29.5	28	2	A-B
An et al., 2022 [27]	clinical trial	40	42.3	23	17	C-D
Villiger et al., 2017 [28]	clinical trial	12	60.0	NA	NA	C-D
An et al., 2018 [29]	clinical trial	10	44.2	6	4	C-D
Wall et al., 2015 [30]	interrupted time series design	5	58.6	5	0	D
Duffell et al., 2019 [31]	clinical trial	11	41.5	10	1	C-D
Villiger et al., 2013 [32]	pretest–posttest design	14	52.7	9	5	C-D
Villiger et al., 2015 [33]	longitudinal pilot study	9	55.1	5	4	D
Lee et al., 2021 [34]	clinical trial	20	55.1	13	7	C-D
Maresca et al., 2018 [35]	case report	1	60.0	1	0	NA
Miguel A. L. Nicolelis et al., 2022 [36]	clinical trial	8	30.1	8	0	A
van Dijsseldonk et al., 2018 [37]	pretest–posttest design	15	59.0	11	4	C-D
Zimmerli et al., 2013 [38]	correlational study	12	46.3	9	3	A-B-C-D
Michibata et al., 2021 [39]	case report	1	41.0	0	1	A
Maggio et al., 2023 [40]	case–control study	42	58.6	22	20	A-B
Azurdia et al., 2022 [41]	concurrent nested design	11	43.3	5	6	NA
Trost et al., 2022 [42]	clinical trial	27	45.8	22	5	A
Austin et al., 2020 [43]	clinical trial	16	54.3	16	0	A-B-C-D
Lakhani et al., 2020 [44]	clinical trial	24	56.20	16	8	A-B-C-D
Putrino et al., 2021 [45]	pretest–posttest design	8	55.0	4	4	B-C-D
Richardson et al., 2019 [46]	clinical trial	59	44.8	47	12	NA
Solcà et al., 2021 [47]	clinical trial	10	47.7	6	4	“others (FBSS)”
Trujillo et al., 2020 [48]	case report	2	45.5	2	0	“others (FBSS)”
Pais-Vieira et al., 2022 [49]	case report	1	52.0	1	0	A
Pozeg et al., 2017 [50]	clinical trial	20	47.3	18	2	A-B-C
M. Gustin et al., 2023 [51]	single-arm study of neuroimaging data	7	45.1	7	0	A
Roosink et al., 2016 [52]	pretest–posttest design	9	53.0	7	2	A-C-D
Shokur et al., 2016 [53]	preliminary study	8	31.1	6	2	A-B
Donati et al., 2016 [54]	clinical trial	8	31.1	6	2	A-B
Jordan et al., 2016 [55]	clinical trial	8	47.5	6	2	A-B-C-D
Sung et al., 2012 [56]	pretest–posttest design	12	28.5	10	2	NA
E. King et al., 2013 [57]	clinical trial	5	40.6	5	0	A-B
Ferrero et al., 2023 [58]	experimental design (not specified)	2	56.5	2	0	B-C
Trincado-Alonso et al., 2014 [59]	clinical trial	15	35.3	11	4	A-B
Tamplin et al., 2020 [60]	experimental design (not specified)	12	51.5	11	1	A-B-C-D

**Table 2:** Upper limb mobility and strength.

Article	Comparative Study	VR Training	Duration of Training (Total Hours)	Tests	Improvement
Lim et al., 2020 [15]	yes	IMMERSIVE	16	MRC grade ASIA-UEMS Grip/pinch strenght BBT NHPT	yes yes yes no no
Al Nattah et al., 2024 [16]	no	NON-IMMERSIVE	N/A	MRC grade Sollerman test	yes yes
Prasad et al., 2018 [17]	yes	NON-IMMERSIVE	6	BBT CUE	no no
Dimbwadyo-Terrer et al., 2016 [18]	yes	IMMERSIVE	7.5	MI	no
Dimbwadyo-Terrer et al., 2016 [19]	yes	SEMI-IMMERSIVE	2	MB NHPT JHFT	no no no
Casadio et al., 2011 [20]	no	BMI/BCI + VR	N/A	MMT	yes
Bressi et al., 2023 [21]	no	SEMI-IMMERSIVE	15	Hand force assesment	yes

MRC grade: medical research council muscle power scale; ASIA UEMS: The American Spinal Injury Association Impairment Scale; BBT: Box and Block Test; NHPT: Nine Hole Peg Test; CUE: Capabilities of Upper Extremity (CUE) questionnaire; MI: The UL part of Motricity Index; MMT: modified manual muscle test; MB: Muscle Balance; JHFT: Jebsen Taylor Hand Function.

**Table 3:** Lower limb mobility, gait and walking, postural stability, and balance.

Article	Comparative Study	VR Training	Duration of Training (Total Hours)	Tests	Improvement
Nair et al., 2022 [22]	yes	NON-IMMERSIVE	6	MFRT T-shirt test	yes no
Goel et al., 2021 [23]	yes	IMMERSIVE	15	MFRT FIST	yes yes
Manzanares et al., 2021 [24]	no	SEMI-IMMERSIVE	10.5	MFRT	no
Sengupta et al., 2020 [25]	yes	SEMI-IMMERSIVE	7.5	MFRT BBS POMA-B	no no no
Khurata et al., 2017 [26]	yes	NON-IMMERSIVE	15	MFRT T-shirt test	yes yes
An et al., 2022 [27]	yes	IMMERSIVE	6	CST TUG 10MWT	yes yes yes
Villiger et al., 2017 [28]	no	SEMI-IMMERSIVE	11.25	TUG BBS LEMS WISCI II 10MWT 6MWT	yes yes yes no no no
An et al., 2018 [29]	no	SEMI-IMMERSIVE	9	TUG LOS BBS WISCI II ABC	yes yes yes yes yes
Wall et al., 2015 [30]	no	NON-IMMERSIVE	14	FFRT LFRT TUG 10MWT	yes yes no yes
Duffell et al., 2019 [31]	no	SEMI-IMMERSIVE	4	ISNC-SCI	yes
Villiger et al., 2013 [32]	no	SEMI-IMMERSIVE	13.5	BBS LEMS WISCI II 10MWT	yes yes yes yes
Villiger et al., 2015 [33]	no	IMMERSIVE	13.5	BBS LEMS 10MWT	yes yes yes
Lee et al., 2021 [34]	yes	NON-IMMERSIVE	12	FSA LOS	yes yes
Maresca et al., 2018 [35]	no	NON-IMMERSIVE	72	BBS	yes
Miguel A. L. Nicolelis et al., 2022 [36]	no	BMI/BCI + VR	118	ISNC-SCI LEMS	yes yes
van Dijsseldonk et al., 2018 [37]	no	NON-IMMERSIVE	12	2MWT	yes
Zimmerli et al., 2013 [38]	no	NON-IMMERSIVE	0.67	Muscle activity (EMGs)	no
Michibata et al., 2021 [39]	no	IMMERSIVE	13	FACT	yes

MFRT: Modified Functional Reach Test; FFRT: forward functional reach test; LFRT: lateral functional reach test; CST: chair stand test; TUG: timed up-and-go; FIST: Function in Sitting Test; FSA: Force Sensitive Application; LOS: Limit of Stability; BBS: Berg Balance Scale; POMA-B: balance section of the Tinetti Performance-Oriented Mobility Assessment; ISNC-SCI: bilateral International Standards for Neurological Classification of SCI; LEMS: Lower Extremity Motor Score; WISCI II: the Walking Index for Spinal Cord Injury; 10MWT: 10 m walking test; 6MWT: 6 m walking test; 2MWT: 2 m walking test; ABC: Activities-Specific Balance Confidence; FACT: Functional Assessment of Control of Trunk.

**Table 4:** Quality of life, daily life independence, functional/cognitive ability, and psychological outcomes.

**Article**	**Comparative Study**	**VR Training**	Duration of Training (Total Hours)	Tests	Improvement
Lim et al., 2020 [15]	yes	IMMERSIVE	16	SCIM III/K-SCIM/SCIM-SR	no
Prasad et al., 2018 [17]	no	NON-IMMERSIVE	6	WHOQOL-BREF SCIM III/K-SCIM/SCIM-SR	yes yes
Dimbwadyo-Terrer et al., 2016 [18]	yes	IMMERSIVE	7.5	SCIM III/K-SCIM/SCIM-SR FIM BI	no no no
Dimbwadyo-Terrer et al., 2016 [19]	yes	SEMI-IMMERSIVE	2	SCIM III/K-SCIM/SCIM-SR BI	yes yes
Goel et al., 2021 [23]	yes	IMMERSIVE	15	SCIM III/K-SCIM/SCIM-SR	no
Manzanares et al., 2021 [24]	no	SEMI-IMMERSIVE	10.5	SCI QL-23 SCIM III/K-SCIM/SCIM-SR	yes yes
Khurana et al., 2017 [26]	yes	NON-IMMERSIVE	15	SCIM III/K-SCIM/SCIM-SR	yes
Villiger et al., 2017 [28]	no	SEMI-IMMERSIVE	11.25	SCIM III/K-SCIM/SCIM-SR	yes
Duffell et al., 2019 [31]	no	SEMI-IMMERSIVE	4	SCIM III/K-SCIM/SCIM-SR	yes
Villiger et al., 2013 [32]	no	SEMI-IMMERSIVE	13.5	SCIM III/K-SCIM/SCIM-SR	yes
Villiger et al., 2015 [33]	no	IMMERSIVE	13.5	SCIM III/K-SCIM/SCIM-SR	yes
Maresca et al., 2018 [35]	no	NON-IMMERSIVE	72	MoCA CAM TMT RAVLI/RAVLR Weigl's sorting test Raven coloured matrices VFT SFT FIM HRS-D HRS-A	yes yes yes yes yes yes yes yes yes yes yes
Maggio et al., 2023 [40]	yes	SEMI-IMMERSIVE	24	SF12 MoCA BDI	yes yes yes
Azurdia et al., 2022 [41]	yes	IMMERSIVE	0.1	FSS/FAS	yes
Trost et al., 2022 [42]	yes	NON-IMMERSIVE	1.67	PHQ-9	no
Austin et al., 2020 [43]	yes	IMMERSIVE	2.5	DASS-21	no
Lakhani et al., 2020 [44]	yes	IMMERSIVE	1	PHQ-9 3 feeling intensity scale	yes yes

SF12: The Short Form-12 health status questionnaire; MoCA: Montreal Cognitive Assessment; BDI: The Beck Depression Inventory; FSS/FAS: The Fatigue Severity Scale (FSS), The Fatigue Assessment Scale (FAS); PHQ-9: the Patient Health Questionnaire-9; DASS-21: The Depression Anxiety Stress Scale; BI: The Barthel Index; WHOQOL-BREF: World Health Organization Quality of Life-BREF; SCI QL-23: Spinal cord injury quality of life questionnaire; CAM: attentive matrices test; TMT: trail making test; RAVLI/RAVLR: digit span and the Rey auditory verbal learning immediate and recall; VFT: verbal fluency test; SFT: semantic fluency test; FIM: The Functional Independence Measure; HRS-D: Hamilton rating scale for depression; HRS-A: Hamilton rating scale for anxiety; SCIM III/K-SCIM/SCIM-SR: Spinal Cord Independence Measure III/Korean Spinal Cord Independence Measure/Spinal Cord Independence Measure Self-Report.

**Table 5:** Pain.

Articles	Comparative Study	VR Training	Duration of Training (Total Hours)	Tests	Improvement
Villiger et al., 2013 [32]	no	SEMI-IMMERSIVE	13.5	NPS	yes
Azurdia et al., 2022 [41]	yes	IMMERSIVE	0.1	PSEQ	yes
Trost et al., 2022 [42]	yes	NON-IMMERSIVE	1.67	NRS NPS	yes yes
Austin et al., 2020 [43]	yes	IMMERSIVE	2.5	NRS	yes
Putrino et al., 2021 [45]	no	IMMERSIVE	N/A	NRS	yes
Richardson et al., 2019 [46]	yes	NON-IMMERSIVE	0.33	NRS NPS	yes yes
Solcà et al., 2021 [47]	no	IMMERSIVE	0.125	VAS for neuropathic pain	yes
Trujillo et al., 2020 [48]	no	IMMERSIVE	4.37	VAS for neuropathic pain PCS	yes yes
Pais-Vieira et al., 2022 [49]	no	IMMERSIVE	13.5	VAS for neuropathic pain	no
Pozeg et al., 2017 [50]	no	IMMERSIVE	0.05	VAS for neuropathic pain	no
M. Gustin et al., 2023 [51]	no	IMMERSIVE	1.67	Increased thalamic GABA	yes
Roosink et al., 2016 [52]	no	SEMI-IMMERSIVE	3.0	neuropathic pain intensity (%)	no

PSEQ: The Pain Self Efficacy Questionnaire; NRS: Neuropathic pain intensity Numeric Rating Scale; NPS: Neuropathic Pain Scale; VAS: Visual Analog Scale (for neuropathic pain); PCS: Pain Catastrophic Scale.

**Table 6:** Other outcome measures and feasibility of the VR system.

Articles	Comparative Study	VR Training	Duration of Training (Total Hours)	Tests	Improvement
Villiger et al., 2015 [33]	no	IMMERSIVE	13.5	structural brainstem and brain plasticity	yes
Miguel A. L. Nicolelis et al., 2022 [36]	no	BMI/BCI+VR	118	changes in sensory parameters	yes
Azurdia et al., 2022 [41]	yes	IMMERSIVE	0.1	RPE	yes
Pais-Vieira et al., 2022 [49]	no	IMMERSIVE	13.5	VR feasibility	yes
Pozeg et al., 2017 [50]	no	IMMERSIVE	0.05	VLI FBI	yes no
Roosink et al., 2016 [52]	no	SEMI-IMMERSIVE	3	motor imagery vividness, effort and speed perceived interaction with avatar and virtual environment side effects	yes yes yes
Shokur et al., 2016 [53]	no	IMMERSIVE	0.75	changes in body ownership	yes
Donati et al., 2016 [54]	no	BMI/BCI+VR	1958	changes in sensory parameters	yes
Jordan et al., 2016 [55]	no	NON-IMMERSIVE	N/A	QST	yes
Sung et al., 2012 [56]	no	NON-IMMERSIVE	6	improvement of driving abilities	yes
E. King et al., 2013 [57]	no	BMI/BCI+VR	0.83	use of BCI	yes
Ferrero et al., 2023 [58]	yes	BMI/BCI+VR	0.167	use of BCI	yes
Trincado-Alonso et al., 2014 [59]	no	IMMERSIVE	not pertinent	VR use in diagnostics	yes
Tamplin et al., 2020 [60]	no	IMMERSIVE	N/A	VR feasibility	yes

RPE: Borg’s Rate of Perceived Exertion (RPE); VLI: Virtual Leg Illusion Questionnaire; FBI: full body illusion questionnaire; QST: quantitative sensory testing; BCI: brain–computer interface.

## Data Availability

All data are extractable from the references listed in Table 1.

## Abbreviations

AI	artificial intelligence
AIS	ASIA impairment scale
AR	augmented reality
ASIA	American Spinal Injury Association
BCI	brain–computer interface
BMI	brain–machine interface
CG	control group
EG	experimental group
FBI	full body illusion
LL	lower limbs
ML	machine learning
n.s.	not specified
NPS	Neuropathic Pain Scale
NRS	Numeric Rating Scale
PRISMA	Preferred Reporting Items for Systematic Reviews and Meta-Analyses
SCI	spinal cord injury
UL	upper limbs
VAS	Visual Analog Scale
VR	virtual reality
